# Short- and Long-Term Effects on Physical Fitness in Older Adults: Results from an 8-Week Exercise Program Repeated in Two Consecutive Years

**DOI:** 10.3390/geriatrics10010015

**Published:** 2025-01-16

**Authors:** Manne Godhe, Johnny Nilsson, Eva A. Andersson

**Affiliations:** 1 Department of Molecular Medicine and Surgery, Karolinska Institutet,171 76 Stockholm, Sweden; 2 Department of Physical Activity and Health, The Swedish School of Sport and Health Sciences, 114 33 Stockholm, Sweden; 3 Department of Neuroscience, Karolinska Institutet, 171 77 Stockholm, Sweden

**Keywords:** physiological capacity, exercise, elderly, test-retest, strength, motor fitness, cardiorespiratory fitness

## Abstract

**Introduction**: Information on the long-term maintenance of short-term exercise fitness gains measured by field-based tests is scarce in older adults. This study aimed to investigate short- and long-term changes in various physical fitness parameters after an 8-week exercise program. **Methods**: In this longitudinal study, a total of 265 participants (62% women; mean age 71.4 ± 4.7 years) completed a field-based test battery of 12 fitness tests (22 parameters) at 2 pre-tests and 1 post-test following an 8-week exercise program (2 sessions/week, combining aerobic and strength activities) in 2 consecutive years. The tests assessed muscle endurance, muscle strength, cardiorespiratory fitness, and motor fitness. **Results**: Significant short-term improvements were observed, e.g., in isometric trunk flexion and extension endurance (21–37%) for both sexes in both years. Lower-body muscular endurance improved in the first year (9–12%) for both sexes, while cardiorespiratory fitness (6-min walk test) improved only for men in both years (3%). No changes were seen in submaximal cycle test heart rates or any balance tests in any year. Most fitness parameters did not significantly decrease during the 9-month inter-intervention period, with a few exceptions in trunk strength and walking distance. **Conclusions**: This study demonstrates physical fitness improvements in older adults following short-term exercise interventions and that some of these improvements were maintained long term, whereas a few of these physical fitness test improvements decreased significantly over 9 months in older adults.

## 1. Introduction

The global population is aging, with projections indicating that by 2050, one in six people worldwide will be over the age of 65 [1]. This demographic shift presents significant challenges to healthcare systems and societies, particularly in maintaining the health, independence, and quality of life of older adults. Physical fitness and function play crucial roles in healthy aging, influencing an individual’s ability to perform activities of daily living, maintain social engagement, and reduce the risk of falls and chronic diseases [2,3]. Furthermore, the advantageous cost-efficiency of promoting physical activity in healthcare, especially for the elderly, has previously been reported [4].

The importance of good fitness for older adults’ health has been well established in recent research. A comprehensive meta-analysis demonstrated that higher upper and lower body muscle strength is associated with a lower mortality risk [5]. This finding is further supported by studies showing that poor performance in various physical fitness tests is linked to an increased risk of mortality [6]. Additionally, higher lower-limb strength has also been associated with better health-related quality of life in older adults [7]. The benefits of particularly resistance training include maintaining and/or increasing muscle mass and strength, reducing chronic disease risk and falls, and improving physical function, mobility, daily-life independence, and overall well-being [8]. Furthermore, regular exercise can counteract age-related chronic inflammation, mitigate the effects of metabolic syndrome, and benefit individuals with osteoporosis by increasing bone mineral density and reducing fall-related fracture risk [8].

Regular physical activity and structured exercise programs are widely recognized as effective interventions for improving and maintaining physical function in older adults [9,10,11]. For instance, a meta-analysis by Hurst et al. (2019) demonstrates that same-session combined endurance and strength training can significantly improve both cardiorespiratory and functional fitness in older populations, with interventions ranging from 6 to 24 weeks [12]. The frequency, intensity, and duration of these exercises should be tailored to the individual’s current fitness level and health status, with a general recommendation of at least 150 min of moderate-intensity aerobic activity or 75 min of vigorous-intensity aerobic activity per week, along with muscle-strengthening activities at least twice a week [13].

The length of interventions can vary significantly, with studies showing benefits from short-term programs as brief as 8–12 weeks [14,15], while others demonstrate cumulative benefits from long-term interventions lasting 6–12 months or more [16,17,18]. Understanding the immediate and long-term effects of short-duration exercise programs can be informative in developing effective, sustainable strategies to promote physical fitness in older adults, guide clinical practice in geriatric care, and empower older adults to make informed decisions about their physical activity regimens [19,20].

The present study aims to address this knowledge gap by examining the effects of an 8-week exercise intervention on various physical fitness parameters in older adults, with follow-up assessments in the consecutive year. Specifically, we seek to investigate the immediate effects of the 8-week interventions on muscular endurance, strength, cardiorespiratory fitness, and motor fitness, and the changes of these effects over a one-year follow-up period. Furthermore, two self-reported questions on exercise habits were included to analyze changes in physical activity throughout the two-year study period.

## 2. Materials and Methods

### 2.1. Study Design

**Figure 1:**
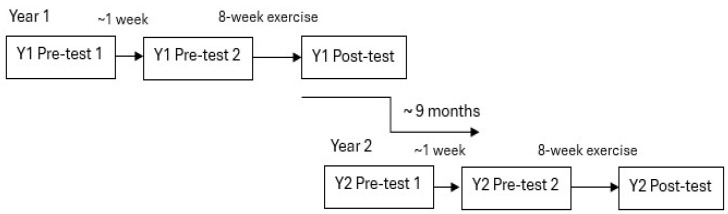
Schematic description of the study design.

In this longitudinal study, 12 field-based fitness tests (yielding 22 test parameters) were conducted on 3 occasions each year: before the exercise intervention (Y1 pre-test-1), 1 week later (Y1 pre-test-2), and after the 8-week exercise intervention (Y1 post-test) in the first year (see Figure 1). This testing sequence and exercise intervention was repeated in the same period one year later: the first pre-test (Y2 pre-test-1), the second pre-test one week later (Y2 pre-test-2), and after the eight-week exercise intervention (Y2 post-test) (see Figure 1). All testing and exercise sessions were supervised by health promotion bachelor program students from The Swedish School of Sport and Health Sciences, who had completed comprehensive coursework in aerobic exercises, muscle strength training, anatomy and physiology, and fitness test methodology, including anthropometric measurements.

### 2.2. Participants

Participants were recruited from a few municipalities in the Greater Stockholm area (mainly from the Solna municipality). Recruitment strategies included advertisements in local media outlets, social media platforms, and targeted placement of promotional materials in locations frequently visited by senior citizens. Inclusion criteria for the study were that the participants were aged 65 years or older, able to walk independently without assistive devices, living independently in their own homes, willing and able to participate in all study components, including two pre-intervention physical fitness tests, an 8-week supervised exercise intervention, and a post-intervention physical fitness test, and were available to repeat the entire protocol (pre-tests, intervention, and post-test) during two consecutive spring semesters. Nine older women did not meet the inclusion criteria due to missing data from one or more test occasions. The exclusion criteria were severe sickness, such as heart failure and severe joint disease. No participants were excluded from the study due to sickness.

The health program coordinator in each municipality collected volunteers each year and relayed the contact information of the health project. Students contacted their assigned seniors and scheduled time at the beginning of the spring term for a joint group introductory meeting and physical fitness testing. Furthermore, before the project, the municipal health program coordinator contacted local training centers and gyms where the older adults were able to exercise free of charge during the 8-week exercise program. Between the 8-week exercise program in year one and year two, no organized training was performed within the study.

The study was approved by the Stockholm regional ethics committee (ID: 2017/2064-32, 2021-00948). All participants were informed about the study in advance and gave their written informed consent to participate each year and a health declaration was signed before each test occasion.

### 2.3. Exercise Period

The participants were randomly assigned to exercise subgroups of 10–30 participants and performed 60-min student-led exercise sessions twice weekly for 8 weeks throughout March and April. The exercise sessions comprised aerobic gymnastics and circuit training, targeting major muscle groups through aerobic and strengthening activities preceded by 5–10 min of warm-up. Balance exercises were incorporated for about 5 min, with the final portion dedicated to low-intensity exercises and relaxation. The circuit training involved 8–10 exercises, with about 8–12 repetitions per set in 2–3 sets. Exercise intensity ranged from “somewhat hard” to “hard”, corresponding to 60–85% of one’s maximum aerobic capacity. For further exercise descriptions, see Godhe et al., 2024 [21].

### 2.4. Test Procedures

Measurements were conducted at The Swedish School of Sport and Health Sciences or a municipal gym for approximately two hours, including rest periods between tests. The same test leader guided participants through a battery of tests on three separate occasions where the tests were administered in the same order each time. Participants were encouraged during the test performances; there was no familiarization preceding the first session, pre-test 1.

**Figure 2:**
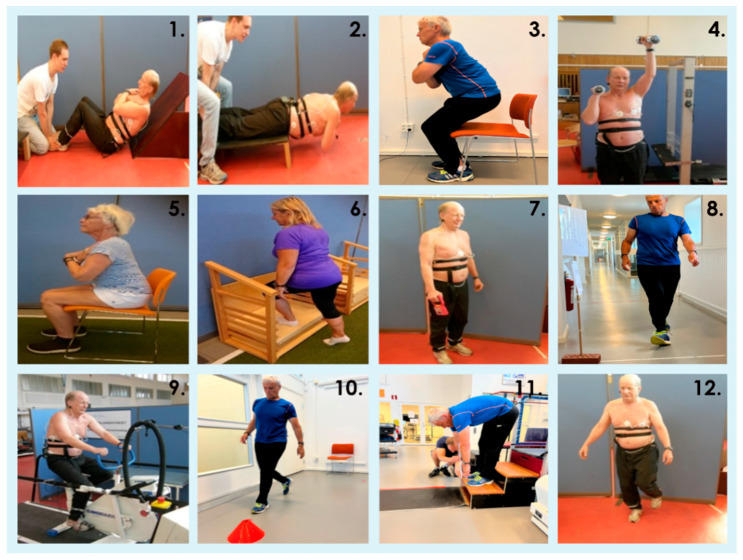
The images illustrate the field fitness tests studied: isometric trunk flexion endurance (45° in the hip joint); (**1**), isometric trunk extension endurance (**2**), 50 sit-to-stands (50 sit-to-stand speed; number of successful chair-bounces (n out of 50)); 30-s sit-to-stand; (**3**), alternating shoulder presses (**4**), five sit-to-stands (sitting on chair between each stand-up); (**5**), maximal step-height test (MST), left and right leg, respectively (**6**), handgrip strength (left and right); (**7**), 6-min walk test (6MWT); (**8**), Ekblom-Bak cycle ergometer test (measuring HR (heart rate) during two workloads); (**9**), time-up-and-go (TUG; **10**), stand-and-reach (**11**), and one-leg standing balance test (left and right, open and closed eyes); (**12**). Figure from Godhe et al., 2024, in the journal Gerontology [21].

Anthropometric measurements included height, weight, BMI, waist circumference, and body composition assessed through bioimpedance recordings (Tanita BC-418MA, Tanita Corporation, Arlington Heights, IL, USA). Tests included measures of muscular endurance, muscular strength, cardiorespiratory fitness, and motor fitness. A total of 12 physical fitness tests were conducted (shown in Figure 2 and described below), yielding 22 test parameters. All fitness test assessments for each participant across the three test occasions in year 1 and year 2 were incorporated into the analyses. However, if an individual was unable to perform a specific fitness test despite completing all other tests, such as the shoulder press, due to shoulder pain on one test occasion, only that test was excluded from the analyses. Consequently, all six test occasions within years one and two for that participant were excluded from analysis for that specific fitness test, regardless of whether only one occasion (e.g., only post-test) was affected.

#### 2.4.1. Muscular Endurance Tests

1. Isometric trunk flexion at 45° was assessed in seconds and performed for as long as possible while seated on the floor, with the trunk held statically backward at 45° (arms crossed over the chest, knees bent 90°) with the ankles supported by the test leader. A wooden 45° frame was positioned just behind the participant as a reference angle [21].
2. Isometric trunk extension (Sorensen’s test, assessed in seconds) was performed prone for as long as possible while holding the trunk horizontally (crossed arms on the chest) and the lower body on a bench (iliac crest at the bench edge, with the test leader supporting the ankles). Timing started, in these two endurance tests, when the correct trunk position was reached and was stopped at exhaustion [22].
3. 30 s sit-to-stand (n out of 50): The participants went from sitting to standing 50 times as fast as possible, being instructed to only touch the chair seat if possible. Otherwise, a complete sit-down was performed between each rise (46 cm chair height). Time and speed/frequency (number of sit-to-stands/second) were noted. In case 50 sit-to-stands were not completed, the number of completed sit-to-stands was used to calculate the speed. Furthermore, all completed sit-to-stands during the first 30 s were noted in the test (for 30 s sit-to-stand, see also [23]). The number of sit-to-stands (n of 50) where the chair seat was briefly bounced on was also noted [21].
4. Shoulder press: Hand weights (5 kg for men and 3 kg for women) were alternately pressed from shoulder height to straight arm (cadence 1/s) until exhaustion. When the participant could not perform another press or keep cadence rhythm, the test was stopped. The number of shoulder presses was noted [21].

#### 2.4.2. Muscular Strength Tests

5. Five sit-to-stands: Time in seconds was measured while performing five sit-to-stands as fast as possible and sitting down fully on the 46-cm high chair between each raise, with start and stop in a seated position [23].
6. Maximal step height test (MST): On either leg, a standardized MST was performed in 3-cm increments on a specially designed step-up box until the highest height was reached. Correct body posture was required, without support from hands or the other leg [24].
7. Grip strength: A hand-grip dynamometer (Sagitta, Sagitta Pedagog AB, Mariestad, Sweden) was squeezed as hard as possible for 4–5 s while standing, with arms and hands hanging. Three measurements were performed on each hand alternately with a short rest between trials. The highest grip strength from the three trials was noted (Nm) [25].

#### 2.4.3. Cardiorespiratory Fitness Tests

8. Six-minute walk test (6MWT): Participants walked as fast as possible around a horizontal/flat course (in ~50 m-laps) indoors for 6 min, after which the distance was noted [23].
9. Submaximal cycling test: Participants cycled with a cadence of 60 rpm (revolutions per minute) during two 4-min periods (according to the Ekblom-Bak cycling protocol [26]), first at a standardized work rate of 32 watts and second at an individually determined rate of work (yielding a perceived-exertion rate (RPE) of ~14, based on sex and training background). Generally, men had higher workloads, although, to enable comparisons between tests, the same individual workload was used on all three test occasions. Mean HR was calculated for the last minute (with time marks of 3.15, 3.30, 3.45, and 4.0 min) at both work rates. HR was measured with a Polar 400 (Electro Oy, Kempele, Finland) as heartbeats per minute (bpm).

#### 2.4.4. Motor Fitness Tests

10. Timed up-and-go (TUG): From a sitting position (46-cm chair height), participants walked as fast as they found comfortable 3 m around a floor mark and returned to sitting [23].
11. Stand-and-reach: Participants, standing with straight legs on a step, reached down toward their toes. The distance from their fingertips to their feet was recorded as ± cm, above/below feet [21].
12. One-leg balance test: With open eyes, on either leg, a balance test was conducted for 60 s. The amount of support needed with the non-balancing foot or arm (n/60 s) and the time to first use support (1st miss) was noted. One-leg balance tests with closed eyes were timed until the first support was needed (s) [21].

Some of the physical fitness tests were introduced later in the health project, which explains the lower number of participants (n) for those tests. This was especially true for the maximal step-up test (MST) and, for example, the 50 sit-to-stands and Ekblom-Bak cycle ergometer test (for test descriptions, see below). Tests such as the isometric trunk flexion, shoulder press, step-up height, and rise-up time were novel for evaluating older adults.

### 2.5. Survey Questions

Before and after the 8-week exercise period (both years 1 and 2), the participants answered two questions about their physical exercise habits: “Have you changed your physical exercise habits in the last six months?”. The five fixed response options were: “Increased a lot”, “Slightly increased”, “As before”, “Reduced slightly”, and “Reduced a lot”. In the subsequent analyses, these responses were scored from 1 up to 5, where 5 represented the response “Increased a lot” and 1 represented “Reduced a lot”.

The other question asked: “How many days per week are you usually physically active for at least 30 min in total? (at least a fast-walking pace)”. The five response options were: “6–7 days/week”, “5 days/week”, “4 days/week”, “2–3 days/week”, “1 day/week”, and “0 day/week”. The responses were scored from 1 up to 6, where 5 represented “5 days/week” and 1 represented “0 day/week”.

### 2.6. Statistics

Descriptive data were presented as mean scores and standard deviation (±SD). The physical fitness test data generally followed a normal distribution. A repeated measures ANOVA (RM-ANOVA) was conducted on the six test occasions to analyze significant time effects and interactions (time x sex effects). Bonferroni’s post hoc tests were used to determine significant differences from pre-test 2 to post-test, both years, and from Y1 post-test to Y2 pre-test 2. The significance threshold for RM-ANOVA was set at *p* < 0.05, with adjustments for multiple testing (*p* < 0.0125). The effect size over time in the RM-ANOVA was analyzed using partial eta square (where 0.01 indicates a small effect, 0.06 indicates a moderate effect, and 0.14 indicates a large effect). For analysis of the survey questions, a Wilcoxon signed-rank test was performed. Statistical analyses were performed using Jamovi (Version 2.4, Computer Software).

## 3. Results

### 3.1. Participants

A total of 265 older adults (102 men, 163 women) participated in this study. The mean age was 71.6 years for men and 71.3 years for women at year 1 testing. Anthropometric data are presented in Table 1.

### 3.2. Intervention Results

Table 2 presents mean values from the pre-2 and post-test sessions in years 1 and 2. No difference in changes in physical fitness between the sexes was seen over time (generally, no significant interaction effect time x sex was found). Significant differences between pre-2 and post-test scores for all fitness parameters in both years are reported below for men and women separately, along with sex differences at each test occasion (RM-ANOVA with Bonferroni post hoc tests). In addition, the mean difference from the first year’s post-test (Y1 post-test) to the second year’s pre-test 2 (Y2 pre-2) for each parameter (including any significant differences) is shown in Table 2.

Where a significant difference was noted (post exercise for each 8-week intervention period or at 9 months follow-up), the effect sizes (partial eta squared) were predominantly moderate (>0.06) to large (>0.14) (Table 2).

### 3.3. Muscular Endurance

**Figure 3:**
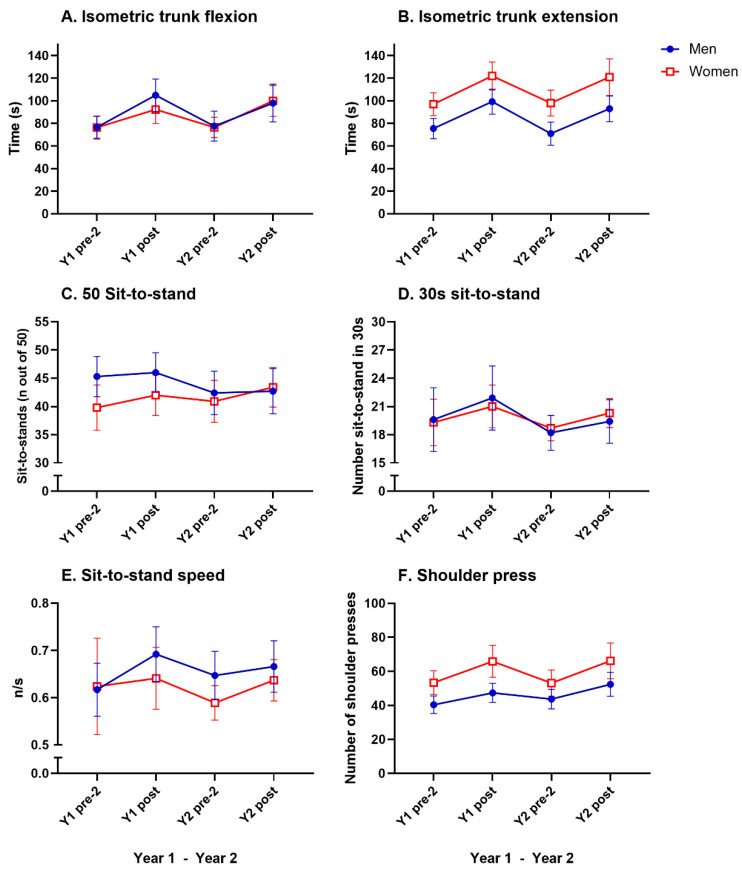
Mean values (with 95% CI) for muscle endurance tests for men and women in absolute values (**A**–**F**). For significant changes, see Table 2.

The RM-ANOVA revealed a significant main effect of time on trunk flexion (F(5, 235) = 23.07, *p* < 0.0001, η^2^p = 0.089) and for trunk extension (F(5, 210) = 33.20, *p* < 0.0001, η^2^p = 0.137). Significant improvements were observed in the post-hoc Bonferroni test from pretest-2 to post-test for isometric trunk flexion and extension for both sexes in years 1 and 2 (21–37%; A and B in Figure 3, Table 2). A significant main effect of time was seen in the shoulder press (F(5, 219) = 15.74, *p* < 0.0001, η^2^p = 0.067). However, the post-hoc Bonferroni test showed that the shoulder press only improved significantly for women (23–25%, F in Figure 3, Table 2) in Y1 pre-test 2 to Y1 post-test, but not in year 2.

A significant main effect of time was seen in the 30 s sit-to-stand (F(5, 68) = 6.38, *p* < 0.0001, η^2^p = 0.086). Bonferroni post hoc analyses showed significant improvements from pre-test 2 to post-test for both sexes for both years 1 and 2 (9–12%, D in Figure 3, Table 2). A significant main effect of time was seen in the 50 sit-to-stand speed test (F(5, 130) = 2.97, *p* < 0.0116, η^2^p = 0.022). Bonferroni post hoc analyses showed a significant improvement for women from pretest 2 to post test in year 2 only (8%, E in Figure 3, Table 2).

Post hoc analyses revealed significant decreases between Y1 posttest and Y2 pretest 2 in isometric trunk flexion in men (−26%, A in Figure 3, Table 2) and isometric trunk extension in both sexes (−20–29%, B in Figure 3, Table 2).

No significant sex differences were observed in muscular endurance tests. Multiplying the number of shoulder presses by the weight resulted in women completing 73–83% of the volume values compared to men.

All muscle endurance results are presented in Table 2 and in Figure 3.

### 3.4. Muscular Strength

**Figure 4:**
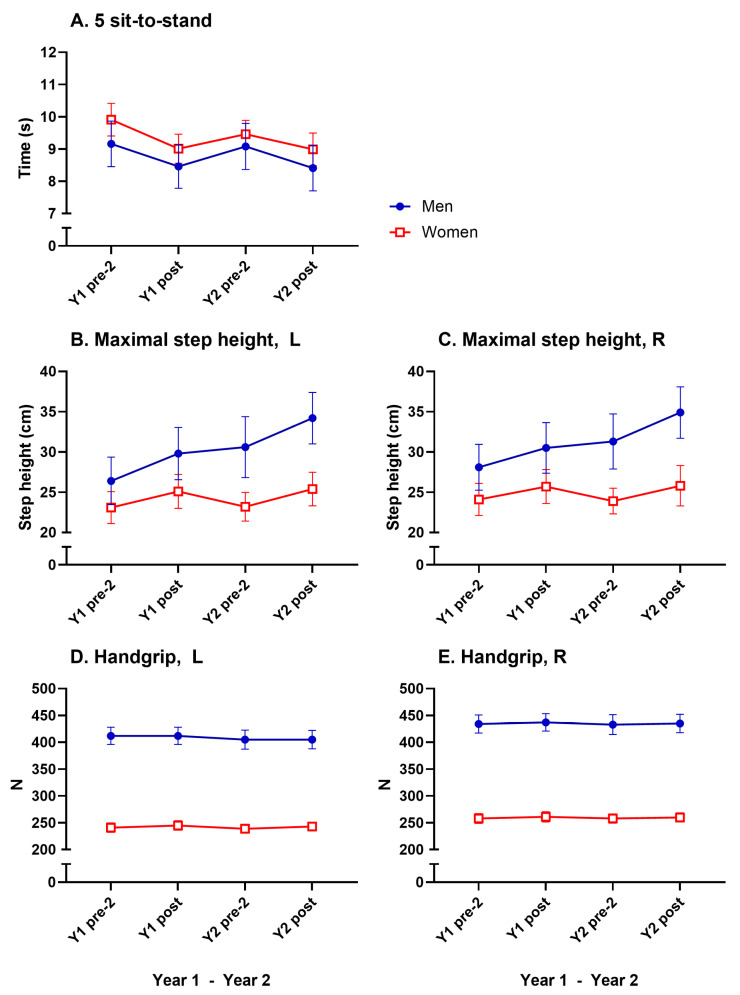
Mean values (with 95% CI) for muscle strength tests for men and women in absolute values (**A**–**E**). For significant changes, see Table 2.

A significant main effect of time was seen in the five sit-to-stands (F(5, 188) = 16.31, *p* < 0.0001, η^2^p = 0.080). Bonferroni post hoc analyses showed significant improvements from pre-test 2 to post test for both sexes in both years (8–9%, Figure 4A, Table 2). A significant main effect of time was seen in MST left (F(5, 59) = 9.64, *p* < 0.0001, η^2^p = 0.140). Bonferroni post hoc analyses showed significant improvements in MST left from pre-test 2 to post test for men in both years (12–13%, Figure 4B, Table 2). No significant improvements were seen in handgrip strength for either sex in either year (Figure 4D,E, Table 2).

No significant decreases for any muscle strength test were noted between Y1 post test and Y2 pre-test 2.

A significant main effect of sex was noted in MST right (F(1, 59) = 21.8, *p* < 0.0001, η^2^p = 0.269) and in MST left (F(1, 59) = 19.4, *p* < 0.0001, η^2^p = 0.248). Bonferroni post hoc tests showed significant sex differences in Y2 pre-test 2 and Y2 post-test in both legs (women had 74–88% of the men’s values). A significant main effect of sex was noted in handgrip right (F(1, 236) = 431, *p* < 0.0001, η^2^p = 0.646) and handgrip left (F(1, 236) = 423, *p* < 0.0001, η^2^p = 0.642). Bonferroni post hoc tests showed significant sex differences on all test occasions in years 1 and 2 for both hands (women 58–60% of the men’s values). No significant sex differences were observed in the five sit-to-stands. All muscle strength results are presented in Table 2 and in Figure 4.

The step height test showed a significant interaction time x sex effect, where the men’s score increased significantly higher than in women from Y1 pre-test 2 to Y2 post-test in the left leg (F(5, 59) = 3.71, *p* < 0.0028, η^2^p = 0.059). The corresponding value for the right leg was (F(5, 59) = 2.56, *p* < 0.0273, η^2^p = 0.042).

### 3.5. Cardiorespiratory Fitness

**Figure 5:**
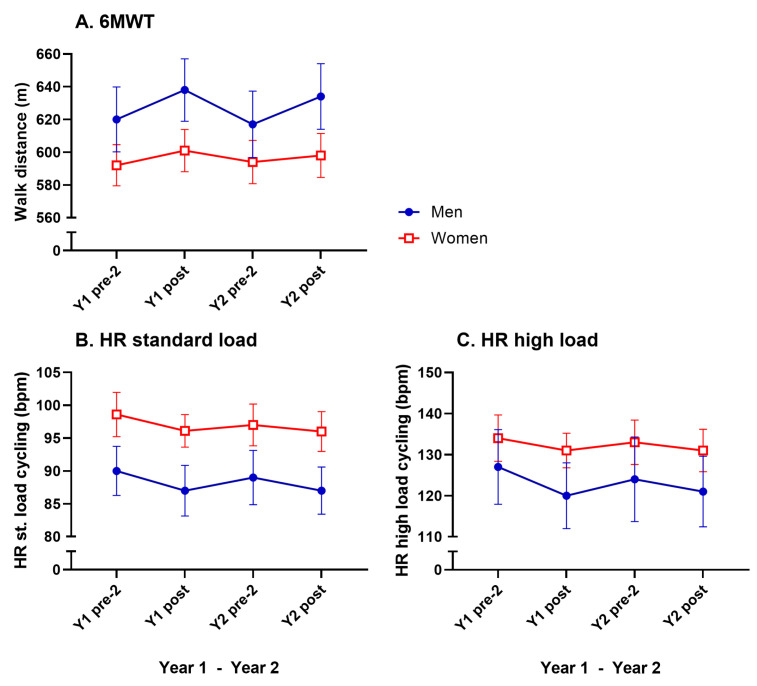
Mean values (with 95% CI) for cardiorespiratory fitness tests for men and women in absolute values (**A**–**C**). For significant changes, see Table 2.

A significant main effect of time was seen in the 6MWT (F(5, 239) = 13.80, *p* < 0.0001, η^2^p = 0.055). Bonferroni post hoc analyses showed significant improvements from pre-test 2 to post test in men only for both years (3%, Figure 5A, Table 2). No significant improvements were seen in heart rate during cycling at standard or high loads (Figure 5B,C, Table 2).

Bonferroni post hoc analyses showed a significant decrease between Y1 post test and Y2 pre-test 2 in the 6MWT for men (−3%, Figure 5A, Table 2).

No significant sex differences were noted in the 6MWT and standard load cycling. Comparison between sexes for the high-load cycling heart rate was not appropriate due to the generally higher loads for men. All cardiorespiratory fitness test results are presented in Table 2 and in Figure 5.

### 3.6. Motor Fitness

A significant main effect of time was seen in TUG (F(5, 193) = 12.75, *p* < 0.0001, η^2^p = 0.062). Bonferroni post hoc analyses showed a significant improvement from Y2 pre-test 2 to Y2 posttest in women (2.6%, Figure 5A, Table 2). A significant main effect of time was seen in the stand-and-reach tests (F(5, 250) = 10.32, *p* < 0.0001, η^2^p = 0.040). Bonferroni post hoc analyses showed significant improvements from Y2 pre-test 2 to Y2 post-test 2 in women (−1.4 cm, Figure 5B, Table 2). No significant improvements were seen in the balance tests for either sex in either year (6C, 6D, 6E, 6F, 6G, 6H).

No significant decreases for any motor fitness test were noted between Y1 posttest and Y2 pre-test 2.

A significant main effect of sex was observed in the stand-and-reach tests (F(5, 250) = 55.4, *p* < 0.0001, η^2^p = 0.181). Bonferroni post hoc analyses showed a significant difference on all test occasions in both years.(~10 cm). No significant sex differences were noted in TUG or balance tests.

**Figure 6:**
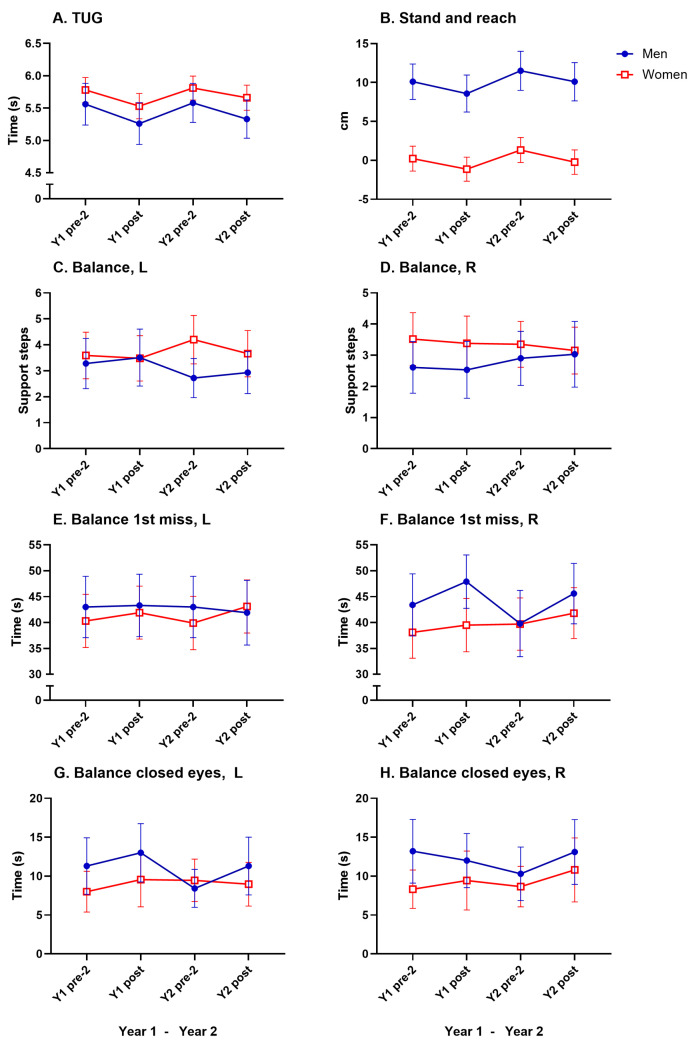
Mean values (with 95% CI) for motor fitness tests for men and women in absolute values (**A**–**H**). For significant changes, see Table 2.

All motor fitness test results are presented in Table 2 and in Figure 6.

### 3.7. Survey Question About Exercise Habits

Overall, 78% of the older adults in this study ticked any of the three following answers: “Increased a lot”, “Slightly increased”, or “As before” (just before the start of the 8-week exercise period in year two) for the question: “Have you changed your physical exercise habits in the last six months?”. This means that 22% of all men and women answered the two other options indicating decreased exercise habits: “Reduced slightly” or “Reduced a lot”.

The mean ratings of exercise habits significantly increased (*p* < 0.001) from before to after the 8-week exercise period from 2.9 to 3.6 in year 1 and from 2.9 to 3.4 in year 2 for men, and similarly for women in year 1 from 3.0–3.8 and from 2.9 to 3.6 in year 2. After the 9-month inter-intervention period, before the exercise period in year 2, a significant decrease in the rating (*p* < 0.001) was noted for both men (from 3.6 to 2.9) and women (from 3.8 to 2.9) when the rating changed in their physical exercise habits in the last six months.

The mean ratings and % reporting 5 days/week or higher in the question “How many days per week are you usually physically active for at least 30 min in total? (at least a fast-walking pace)” were 3.4 (17%) and 3.8 (24%) for men (*p* < 0.01) and 3.9 (34%) and 4.4 (49%) for women (*p* < 0.01) in year 1, before and after 8-week exercise, respectively. Corresponding values in year 2 were 3.6 (28%) and 3.8 (27%) for men (ns) and 4.0 (33%) and 4.2 (43%) for women (*p* < 0.01), respectively. The decrease in the ratings from Y1 post test to Y2 pre-test 2 (i.e., in the follow-up period) was 3.8 (24%) to 3.6 (28%) for men (ns) and 4.4 (49%) to 4.0 (33%) for women (*p* < 0.01).

## 4. Discussion

This study examined the short- and long-term effects of an 8-week exercise program on various physical fitness parameters in older adults over two consecutive years, providing insights into changes in physical fitness post exercise and in the subsequent year. Our findings demonstrate significant improvements across multiple fitness domains following the short-term interventions, with many benefits maintained during the 9-month follow-up period. Furthermore, no significant interaction effects of the changes between men and women over the two years were seen (time x sex), except in the maximal step height test (MST).

### 4.1. Intervention Results

#### 4.1.1. Muscular Endurance and Strength

There was a substantial improvement in isometric trunk flexion and extension strength for both men and women across both years of the intervention. These gains, ranging from 21–37% with moderate to large effect sizes (partial eta squared ranging from 0.118 to 0.381), suggest that the short exercise program was particularly effective in enhancing core strength endurance. Our results are similar to the improvements of 20–37% in isometric endurance strength in trunk flexion and trunk extension in older adults (65–79 yrs) who performed the 8-week exercise intervention for the first time [21]. Similarly, results in one study saw increases of 21–34% in maximal trunk flexion and extension torque (Nm) [27]. Core strength has previously been linked with functional performance and reducing fall risk in older adults [28]. However, field fitness tests for abdominal and back strength in older adults are very scarce or absent in evaluations of exercise interventions [23].

The improvements in the 30 s sit-to-stand test observed in the first year (9–12% for both sexes) and not the second year suggest that initial gains in lower body muscular endurance may plateau over time in this test parameter. However, large effect sizes (partial eta squared of 0.348 for men and 0.342 for women and 0.197 and 0.283 in year 1 and year 2, respectively) were observed.

Interestingly, significant decreases were noted in some muscular endurance measures between the end of year 1 and the start of year 2, particularly in trunk flexion for men (−26%) and trunk extension (both sexes, 20–29%), and also in shoulder press for women (−19%). This decline could be attributed to a potential detraining effect during the break (of about 9 months) between intervention years, emphasizing the importance of consistent, year-round exercise for maintaining muscular endurance in older adults [29].

#### 4.1.2. Cardiorespiratory Fitness

The 6-Minute Walk Test (6MWT) showed significant intervention improvements in men both during the first and the second year; effect sizes were large (with partial eta squared results of 0.170 and 0.174 for years 1 and 2, respectively). The 6MWT was one of the few included tests that showed a significant decrease, only for men, in the follow-up analysis 9 months later. These results indicate a reduction in aerobic fitness in the detraining period for older men, as a significant correlation has been shown between 6MWT distance and VO2max scores with direct oxygen consumption measures in a maximal cycle ergometer test for men only [30]. However, in the present study, no significant changes were observed in heart rate submaximal cycling tests (Ekblom-Bak test) [26] for either year in men or women, indicating that aerobic capacity may require more prolonged or intense interventions to show significant improvements, especially for older women, presenting no significant intervention improvement in any of the aerobic fitness test parameters in this study.

#### 4.1.3. Motor Fitness

The Timed Up and Go (TUG) test showed significant improvements for women in the second year, while both men and women improved in the stand-and-reach test in the same period with small to large effect sizes (partial eta squared ranging from 0.048 to 0.183). No significant changes were observed in balance tests, indicating that balance may require more targeted or prolonged interventions to improve.

Time decreases in the TUG of 0.1–0.3 s in this study were lower than those reported in a meta-analysis reporting −0.92 s derived from 28 studies [31] and were similar to Gonçalves et al., 2021, where the time decreased significantly with 0.16 s [29]. Our results of higher flexibility, being able to reach 1.4–1.5 cm further from standing, are lower than the reported 2.9 cm further reach in the sit-and-reach test after a 9-month exercise intervention (combined exercise, 2/week) [29]. Our results in the TUG, one-legged stand (open and closed eyes), and stand-and-reach tests were similar to the results in previous reports [21,32,33].

#### 4.1.4. Sex Differences

The men and women in this study showed similar changes over time. No significant interaction effects (time x sex) were found for any test parameter except for MST, where men showed a significantly higher increase than women from pre-2 year 1 to the post-test year 2 (left leg *p* = 0.003, right leg *p* = 0.027).

We saw an improvement in the 6MWT aerobic test only for men from pre to post test in both years. Interestingly, a meta-analysis reported greater pre-post exercise effects in older women for motor fitness, and conversely, greater upper and lower body strength and cardiorespiratory fitness for older men ≥ 60 years [34].

Comparing scores between men and women on all four test occasions, significant sex differences were observed in the strength measures (e.g., step height, handgrip strength) and flexibility solely (stand-and-reach), in line with previous reports [21,25].

The observed sex differences in certain strength measures (e.g., MST, handgrip strength) and flexibility (stand-and-reach) highlight the importance of considering gender-specific responses to exercise interventions in older adults. These differences may reflect varying baseline fitness levels, physiological responses to exercise, or potentially different engagement levels with certain aspects of the program between men and women [8]. However, no significant sex differences were observed in muscular endurance tests, TUG, or balance tests.

#### 4.1.5. Long-Term Maintenance of Benefits

Studies of long-term maintenance following short-term intervention (8-week period) are scarce. The follow-up data from two consecutive years of 8-week interventions in our study provide valuable insights into the retention of short-term exercise programs in older adults.

Muscular Endurance: Significant decreases in some muscular endurance measures, evidently in trunk flexion and extension, were observed between the end of year 1 and the start of year 2. This suggests that the initial gains from the 8-week program may not be fully maintained without continued exercise, highlighting the importance of ongoing physical activity for preserving muscular endurance in older adults [29].

Strength measures: The maintenance of improvements in some strength measures (e.g., step height for men, five sit-to-stands, and 30s sit-to-stand for both sexes) over the two-year period is encouraging. It suggests that even a short-term intervention can potentially lead to lasting benefits in certain aspects of muscular strength.

Cardiorespiratory fitness: The limited long-term improvements in cardiorespiratory fitness for men, as measured by the 6-min walk test (6MWT), underscore the need for continuous aerobic exercise to enhance and maintain cardiovascular health in older adults.

Notably, our 9-month follow-up measures generally showed maintained scores across all parameters of endurance, strength, aerobic, and motor fitness tests just before the start of exercise year 2, compared to the post-exercise scores from year 1. Of the 22 physical fitness test parameters, only a few exceptions were noted: isometric trunk flexion and 6MWT distance decreased for men, and isometric trunk extension decreased for both sexes. Importantly, these parameters were again improved by the second year’s 8-week exercise period, reaching similar post-test levels in both years (Table 2).

It is worth highlighting that when comparing post-test scores between years 1 and 2, no significant differences were observed for any physical fitness test variable across sex groups. These results suggest that the natural decline in physical capacities associated with aging could potentially be mitigated to some extent with short-term exercise interventions.

Our findings align with previous research demonstrating that short-term exercise programs can yield significant improvements in various fitness domains, particularly in muscular endurance and strength. Notably, our study showed that most fitness parameters were maintained during the 9-month follow-up period between interventions.

These results are consistent with some previous exercise interventions that have reported maintained fitness scores during follow-up periods in measures of some of the seven field tests within the “Short Physical Performance Battery” (SPPB), originally presented by Rikkli and Jones in 1999 [23]. For instance, Eggenberger et al. (2015) found maintained five sit-to-stand, walking, and balance scores after 1 year of follow-up after 6 months of combined exercise intervention (2/week, n = 47) [35], while Fisher et al. (2018) observed a maintained performance of 30s sit-to-stand, TUG, and 6MWT after 9 months of follow-up of 3 months of combined exercise (3/week, n = 63) [36]. Timmons et al. (2020) found maintained scores in five sit-to-stand, grip strength, TUG, short gait speed, and stair climbing tests; however, leg and chest press decreased 1 year after a 12-week supervised program (combined exercise, 3/week, n = 53) [33]. Likewise, preserved values in five sit-to-stand and grip strength were seen in a 4-month follow-up after 8 weeks with aerobic exercise (3/week, n = 28) [37], and in 30 sit-to-stand, grip strength, arm curl, 6MWT, TUG, and habitual walking speed at a 3-month follow-up in older women (after 12 weeks of resistance/balance exercise, 2/week, n = 68) [38].

However, the literature also includes exercise studies reporting decreased fitness scores over time, particularly with longer follow-up periods. Gomez-Bruton et al. (2020) [39] found impaired scores in the senior fitness test battery after an 8-year follow-up. A lower decline was shown in individuals who continued regular organized physical activity (reported via questionnaires, n = 642). The declines were generally seen for 30 s sit-to-stand, TUG, 6MWT, one-leg balance, arm curl, leg flexibility, and short working (8 years after baseline, n = 642). We also observed some decreases, particularly in isometric trunk flexion and extension and 6MWT, which resonates with these findings. On the other hand, we maintained scores at follow-up for similar tests, as in the senior fitness test battery (e.g., 30 s sit-to-stand, five chair raises, grip strength, TUG, and balance test).

Another longer intervention (combined exercise, 11 months, 2–3/week, n = 60) reported maintained static knee extensor strength one year after the intervention in older adults, whereas the other nine physical fitness parameters declined, e.g., cardiorespiratory fitness, dynamic knee extensor strength (Nm), jump height, 30 s arm curls, and 30 s sit-to-stand [16].

Furthermore, the results from Gonçalves et al. (2021) [29] quite often observed a fitness decrease from post-tests each year to the following pre-test of the 9-month combined exercise (2/week, n = 138) each year (in five SPPB fitness tests, i.e., 30 s sit-to-stand, TUG, arm curl, and 2-min step test); however, improvements from baseline to post-test were shown overall across a 5-year program with annual exercise interventions. Similarly, Gylling et al. (2020) found a decrease in knee extension torque (Nm) in the subgroup that did not continue 1-year heavy resistance training (3/week, n = 265) at 1-year follow-up [40]. These studies reported decreases in certain physical fitness parameters over time, especially in longer follow-up periods. Even a shorter follow-up period of 3 months showed decreased fitness scores; TUG, 30 s sit-to-stand, 6MWT, and grip strength compared to post-exercise scores after 36 weeks of combined exercise (n = 38) [32].

Our study’s unique approach of conducting interventions over two consecutive years allowed us to observe that post-test scores did not significantly differ between years, suggesting a potential mitigation of age-related decline.

These collective findings reinforce the importance of developing strategies to promote consistent exercise engagement among older adults. They also suggest that periodic short-term interventions could help seniors recover from periods of reduced activity, potentially playing a crucial role in maintaining functional independence and quality of life in aging populations.

Research on exercise interventions for older adults consistently emphasizes the importance of maintaining physical activity to preserve fitness gains and mitigate age-related functional decline. The present study, involving 265 seniors and 22 physical fitness test parameters, provides a comprehensive analysis of both short-term and long-term maintenance of 8-week exercise interventions over two consecutive years.

### 4.2. Physical Activity and Physical Fitness Levels in the Participants

The participants in this study reported lower ratings than 5 days per week with at least 30 min of physical activity on all four test occasions. This suggests that most of the community-dwelling older adults in our study did not reach the WHO recommendations with at least 150 min/week with PA of at least moderate intensity [13].

Analysis of self-reported exercise habits revealed that most of the men and women (78%) had either maintained or increased their exercise habits in the last 6 months, as answered before the exercise period in year 2. Thus, only 22% of both sexes responded that they had reduced their exercise levels in the past 6 months. This exercise habit maintenance could be an important long-term outcome of the intervention that requires further investigation, an effect of the exercise program that extends beyond the physical fitness measurements. Between the year-one and year-two 8-week exercise programs, no organized training or exercise advice was given within the scope of this study. It would have been optimal if the physical activity habits were monitored with more objective methods, such as accelerometer measurements. It may have provided a more accurate evaluation of physical activity changes over time and therefore a better understanding of some of the physical fitness changes [41].

The physical fitness levels of the participants in our study compared to previous reports were for: grip strength; slightly lower or similar for both sexes [21,25,33,40], slightly higher or similar [7,21]; 30 s sit-to-stand: similar [21,40,42] or slightly higher [7,16,29,32,39]; five sit-to-stand: similar [21,33], slightly faster [37]; TUG: similar [21,32,33]; 6MWT: similar [21,32], slightly longer [21,36]; submaximal cycle test HR: similar [21]; MST and shoulder press: somewhat lower or similar [21,24]; trunk endurance strength and 50 sit-to-stand speed: similar or somewhat higher [21]; one leg stance, open and closed eyes, stand-and-reach: similar [21].

### 4.3. Strengths and Limitations

This study provides valuable insights into both the immediate and long-term effects of a short exercise intervention on older adults’ physical fitness. However, several limitations should be acknowledged.

One limiting factor is the absence of a control group, which limits our ability to definitively attribute observed changes to the intervention rather than to normal aging processes. However, we can draw some inferences from our previous work [38], which compared a similar 8-week exercise intervention to a control group using accelerometer data. That study demonstrated significantly larger improvements in total physical activity, in time spent at moderate-to-vigorous intensity, and in decreased sedentary time for the intervention group compared to the controls.

An 8-week intervention period with two training sessions per week was chosen for organizational purposes; it had to fit within the course where this health project was part of the student’s educational program. However, previous physical-fitness-evaluated exercise interventions in older adults often have durations of 12 weeks, 8 weeks, or shorter, usually with 2–3 sessions/week [34]. It has previously been reported that exercise should be performed 2–3 times/week in exercise recommendations for older adults [8].

The differential responses observed for some of the test parameters between year 1 and year 2 warrant further investigation. These variations could be due to factors such as individual adaptations to exercise, changes in lifestyle or health status, or variations in program delivery or exercise adherence.

Future research should incorporate more detailed monitoring of exercise adherence and physical activity levels during the follow-up period. This would provide a better understanding of the factors influencing the long-term maintenance of physical fitness improvements. Additionally, exploring the impact of nutrition, sleep, and other lifestyle factors alongside exercise could offer a more comprehensive picture of fitness changes in older adults over time.

A strength of this study lies in its focus on developing and evaluating easy-to-use field fitness tests for older adults in exercise programs. The physical fitness tests used in the present study (including some new field tests) are easy to administer and cost-effective, enhancing the potential for widespread adoption in community-based programs. We demonstrated how short exercise programs can be implemented, cost-free for the participants, in collaboration with municipalities and local training centers. Several factors contributed to these annual short exercise programs being cost-effective. Partly because students led the test and training sessions within their education program. Furthermore, training centers and gyms made their facilities available free of charge for the participants for 8 weeks. Furthermore, various municipal leaders within their services, together with teachers at The Swedish School of Sport and Health Sciences, jointly organized the project. The advantageous cost efficiency of promoting physical activity in health care, especially for older adults, has previously been reported [4]. This can hopefully inspire future health promotion actors to administer this type of program. These results contribute valuable evidence supporting the importance of regular exercise for older adults and emphasize the need for strategies to promote long-term adherence to physical activity in this population. Our experience suggests that older adults often appreciate the ability to track their fitness capacities before and after a structured, supervised exercise period, potentially enhancing adherence to exercise programs.

The present field-based tests used were chosen to avoid ceiling effects as much as possible, e.g., that it is difficult to attain improvements after a certain fitness/exercise level for seniors. Our relatively new physical fitness tests (e.g., isometric trunk endurance tests, shoulder press, 50 sit-to-stand speed, and MST) are designed so that, regardless of physical ability, possible improvements can be made.

Shorter functional tests, such as five sit-to-stands and 30 s sit-to-stands, may not always be as easy to improve if you have high physical fitness at the baseline. We found a significant pre-post improvement for these two latter tests (in both men and women) only for year 1 but not year 2. However, during both intervention years, significant improvements (for both sexes) regarding, for example, trunk strength, shoulder press, MST, and 6MWT were seen. Particularly noteworthy are the improvements observed in various field tests of muscle strength and fitness after an 8-week, twice-weekly exercise program, consistent with our previous findings [21]. These field test results not only provide valuable data for researchers but can also serve as reference values for relatively healthy community-dwelling older adults.

## 5. Conclusions

This study, examining an 8-week exercise intervention conducted over two consecutive spring semesters, demonstrates significant immediate benefits for older adults across multiple fitness domains. Encouragingly, fitness levels were maintained in several test parameters during the 9-month follow-up period.

The findings reveal both similarities and occasional variations in fitness effects between sexes and across the two intervention periods.

In conclusion, this study demonstrates the effectiveness of short-term exercise intervention and provides insights for future research and practical applications in promoting physical fitness among older adults.

## Figures and Tables

**Table 1:** Mean values (±SD) at baseline each year for age, length, weight, BMI, waist circumference, and % body fat for men and women.

	Year 1		Year 2	
Sex	Men (*n* = 102)	Women (*n* = 163)	Men (*n* = 102)	Women (*n* = 163)
Age (yrs)	71.6 ± 4.64	71.3 ± 4.80	72.6 ± 4.64	72.3 ± 4.80
Height (m)	1.78 ± 0.07	1.64 ± 0.05	1.78 ± 0.07	1.64 ± 0.06
Weight (kg)	82.1 ± 11.5	67.1 ± 9.7	81.6 ± 11.2	66.9 ± 9.9
BMI (kg/m^2^)	26.0 ± 3.2	25.0 ± 3.5	25.9 ± 3.1	25.0 ± 3.5
Waist circumf. (cm)	96.8 ± 13.1	86.6 ± 10.9	97.5 ± 9.4	85.4 ± 11.2
Bodyfat (%)	24.6 ± 6.6	34.6 ± 6.1	24.0 ± 7.1	34.3 ± 6.2

**Table 2:** Physical fitness tests with mean values ± SD for men and women at pre-2 and post-test during year 1 and year 2 (i.e., intervention results each year) and follow-up comparisons between the pre-2 II (year 2) and post-test (year 1).

Physical Fitness Test	Sex	n	Y1 Pre-Test 2	Y1 Post-Test	Y1 Post-Test–Y1 Pre-Test 2 (%)	sign.	Partial Eta Squared (ES) Y1 Post-Test–Y1 Pre-Test 2	Y2 Pre-Test 2	Y2 Post-Test	Y2 Post-Test –Y2 Pre-Test 2 (%)	Sign.	Partial Eta Squared (ES) Y2 Post-Test–Y2 Pre-Test 2	Y2 Pre-Test 2–Y1 Post-Test (%)	Sign.	Partial Eta Squared (ES) Y 2 Pre-Test 2– Y1 Post-Test
Isometric trunk flexion	Men	94	76.7 ± 47.5	105 ± 70.6	28.3 (36.9%)	<0.001	0.270	77.7 ± 64.6	98.1 ± 81.4	20.4 (26.3%)	0.007	0.159	−27.3 (−26.0%)	0.002	0.160
	Women	143	76.4 ± 62.6	92.4 ± 74.3	16.0 (20.9%)	0.002	0.118	76.6 ± 54.3	100 ± 82.8	23.4 (30.5%)	<0.001	0.168	−15.8 (−17.1%)	0.122	0.059
Isometric trunk extension	Men	84	75.5 ± 41.1	99.3 ± 50.5	23.8 (31.5%)	<0.001	0.300	71.0 ± 47.1	93.0 ± 52.2	22.0 (31.0%)	0.002	0.330	−28.3 (−28.5%)	<0.001	0.300
	Women	128	97.1 ± 58.0	122 ± 71.6	24.9 (25.6%)	<0.001	0.361	98.1 ± 65.6	121 ± 93.2	22.9 (23.3%)	<0.001	0.142	−23.9 (−19.6%)	<0.001	0.176
Sit to stand out of 50	Men	49	45.3 ± 12.4	46.0 ± 12.3	0.7 (1.5%)	1.000	0.024	42.4 ± 14.8	42.7 ± 15.4	0.3 (0.7%)	1.000	0.000	−3.6 (−7.8%)	1.000	0.036
	Women	66	39.8 ± 16.3	42.0 ± 14.7	2.2 (5.5%)	1.000	0.097	40.9 ± 16.3	43.4 ± 15.4	2.5 (6.1%)	1.000	0.117	−1.1 (−2.6%)	1.000	0.005
30 s sit-to-stand	Men	26	19.6 ± 8.7	21.9 ± 8.6	2.3 (11.7%)	0.007	0.348	18.2 ± 4.8	19.4 ± 6.0	1.2 (6.6%)	0.678	0.197	−3.7 (−16.9%)	0.568	0.206
	Women	44	19.3 ± 8.2	21.0 ± 7.8	1.7 (8.8%)	0.007	0.342	18.7 ± 4.6	20.3 ± 5.2	1.6 (8.6%)	1.000	0.283	−2.3 (−0.11)	1.000	0.113
Sit-to-stand speed	Men	53	0.62 ± 0.20	0.69 ± 0.21	0.08 (12.2%)	0.167	0.319	0.65 ± 0.19	0.67 ± 0.20	0.02 (2.9%)	0.967	0.047	−0.05 (−6.5%)	0.957	0.108
	Women	79	0.62 ± 0.46	0.64 ± 0.29	0.02 (2.7%)	1.000	0.006	0.59 ± 0.16	0.64 ± 0.20	0.05 (8.1%)	0.002	0.161	−0.05 (−8.1%)	0.667	0.035
Shoulder Press	Men	87	40.4 ± 24.0	47.4 ± 26.2	7.0 (17.3%)	0.088	0.279	43.7 ± 26.9	52.4 ± 33.1	8.7 (19.9%)	0.038	0.259	−3.7 (−7.8%)	1.000	0.018
	Women	134	53.4 ± 40.9	65.9 ± 54.8	12.5 (23.4%)	<0.001	0.200	53.1 ± 45.0	66.2 ± 61.6	13.1 (24.7%)	0.001	0.177	−12.8 (−19.4%)	0.029	0.062
Five sit-to-stands	Men	75	9.2 ± 3.1	8.5 ± 3.0	−0.7 (−7.6%)	0.010	0.203	9.1 ± 3.1	8.4 ± 3.0	−0.7 (−7.4%)	0.083	0.124	0.6 (7.3%)	0.500	0.054
	Women	114	9.9 ± 2.7	9.0 ± 2.4	−0.9 (−9.1%)	<0.001	0.214	9.5 ± 2.3	9.0 ± 2.73	−0.5 ( −5.2%)	1.000	0.063	0.5 (0.05)	0.850	0.029
Step height L	Men	24	26.4 ± 7.0	29.8 ± 7.7	3.4 (12.9%)	<0.001	0.478	30.6 ± 8.96	34.2 ± 7.56	3.6 (11.8%)	0.003	0.399	0.8 (2.7%)	1.000	0.006
	Women	37	23.1 ± 5.9	25.1 ± 6.3	2.0 (8.7%)	0.040	0.277	23.2 ± 5.35	25.4 ± 6.26	2.2 (9.5%)	0.048	0.282	−1.9 (−7.6%)	0.970	0.058
Step height R	Men	24	28.1 ± 6.7	30.5 ± 7.5	2.4 (8.5%)	0.043	0.317	31.3 ± 8.10	34.9 ± 7.56	3.6 (11.5%)	0.015	0.520	0.8 (2.6%)	1.000	0.007
	Women	37	24.1 ± 6.0	25.7 ± 6.3	1.6 (6.6%)	0.124	0.217	23.9 ± 4.77	25.8 ± 7.54	1.9 (7.9%)	0.370	0.120	−1.8 (−0.07)	0.971	0.062
Handgrip L	Men	92	412 ± 77.6	412 ± 77.4	0.0 (0.0%)	1.000	0.000	405 ± 86.0	405 ± 82.5	0.0 (0.0%)	1.000	0.000	−7.0 (−1.7%)	1.000	0.023
	Women	146	241 ± 54.1	245 ± 54.5	4.0 (1.7%)	1.000	0.020	239 ± 50.5	243 ± 49.7	4.0 (1.7%)	1.000	0.029	−6.0 (−2.4%)	1.000	0.029
Handgrip R	Men	92	434 ± 81.3	437 ± 78.5	3.0 (0.7%)	1.000	0.004	433 ± 89.0	435 ± 82.5	2.0 (0.5%)	1.000	0.005	−4.0 (−0.9%)	1.000	0.006
	Women	148	258 ± 56.2	261 ± 58.6	3.0 (1.2%)	0.998	0.007	258 ± 52.3	260 ± 52.2	2.0 (0.8%)	1.000	0.006	−3.0 (−1.1%)	1.000	0.004
6MWT	Men	94	620 ± 96	638 ± 93	18 (2.9%)	<0.001	0.170	617 ± 99	634 ± 98	17 (2.8%)	<0.001	0.174	−21.0 (−3.3%)	0.002	0.126
	Women	127	592 ± 77	601 ± 79	9 (1.5%)	0.047	0.072	594 ± 81	598 ± 83	4 (0.7%)	0.966	0.013	−7.0 (−1.2%)	0.731	0.031
HR st.load	Men	46	90.7 ± 12.6	87.7 ± 13.0	−3.0 (−3.3%)	0.413	0.189	89.8 ± 13.9	87.8 ± 12.1	−2.0 (−2.2%)	0.929	0.051	2.1 (2.4%)	0.976	0.034
	Women	72	98.6 ± 14.3	96.1 ± 10.6	−2.5 (−2.5%)	0.399	0.060	97.0 ± 13.7	96.0 ± 13.1	−1.0 (−1.0%)	0.929	0.011	0.9 (0.9%)	1.000	0.011
HR High load	Men	10	127 ± 12.7	120 ± 11.2	−7.0 (−5.5%)	0.546	0.338	124 ± 14.4	121 ± 12.0	−3.0 (−2.4%)	0.952	0.270	4 (3.3%)	0.996	0.152
	Women	27	134 ± 15.3	131 ± 12.6	−3.0 (−2.2%)	0.702	0.134	133 ± 18.2	131 ± 17.4	−2.0 (−1.5%)	0.966	0.058	2 (1.5%)	0.996	0.034
TUG	Men	74	5.6 ± 1.4	5.3 ± 1.4	−0.3 (−5.4%)	0.049	0.125	5.6 ± 1.3	5.3 ± 1.3	−0.3 (−4.5%)	0.032	0.137	0.3 (6.1%)	0.262	0.074
	Women	118	5.8 ± 1.1	5.5 ± 1.1	−0.3 (−4.3%)	0.028	0.107	5.8 ± 1.0	5.7 ± 1.1	−0.1 (−2.6%)	0.006	0.048	0.3 (5.1%)	0.101	0.110
Stand and reach	Men	96	10.1 ± 11.3	8.6 ± 11.7	−1.5 ( − )	0.043	0.085	11.5 ± 12.4	10.1 ± 12.1	−1.4 ( − )	0.004	0.083	2.9 ( − )	0.065	0.185
	Women	157	0.2 ± 10.1	−1.1 ± 9.8	−1.3 ( − )	0.030	0.093	1.3 ± 10.2	−0.1 ± 10.0	−1.4 ( − )	<0.001	0.183	2.4 ( − )	0.023	0.063
Balance L	Men	68	3.3 ± 4.0	3.5 ± 4.5	0.2 (7.0%)	1.000	0.005	2.7 ± 3.6	2.9 ± 3.8	0.2 (7.7%)	1.000	0.008	−0.8 (−22.5%)	1.000	0.017
	Women	113	3.6 ± 4.8	3.5 ± 4.7	−0.1 (−3.1%)	1.000	0.003	4.2 ± 5.5	3.7 ± 5.3	−0.5(−12.9%)	0.610	0.059	0.7 (20.7%)	1.000	0.075
Balance R	Men	59	2.6 ± 3.2	2.5 ± 3.5	−0.1 (−3.1%)	1.000	0.001	2.9 ± 3.6	3.0 ± 4.34	0.1 (4.5%)	1.000	0.004	0.4 (14.6%)	1.000	0.005
	Women	102	3.5 ± 4.3	3.4 ± 4.5	−0.1 (−4.0%)	1.000	0.004	3.4 ± 3.9	3.2 ± 4.1	−0.2 (−6.0%)	1.000	0.010	0 (−0.9%)	1.000	0.017
Balance 1Miss L	Men	54	43.0 ± 21.7	43.3 ± 22.1	0.3 (0.7%)	1.000	0.000	43.0 ± 21.7	41.9 ± 22.9	−1.1 (−2.6%)	1.000	0.004	−0.3 (−0.7%)	1.000	0.000
	Women	78	40.3 ± 22.8	41.9 ± 22.7	1.6 (4.0%)	1.000	0.001	39.9 ± 23.0	43.1 ± 22.9	3.2 (8.0%)	1.000	0.045	−2.0 (−4.8%)	1.000	0.011
Balance 1Miss R	Men	54	43.4 ± 22.0	47.9 ± 18.9	4.5 (10.4%)	1.000	0.060	39.8 ± 23.5	45.6 ± 21.4	5.8 (14.6%)	1.000	0.108	−8.1 (−16.9%)	1.000	0.168
	Women	79	38.1 ± 22.4	39.5 ± 23.0	1.4 (3.7%)	1.000	0.004	39.7 ± 22.6	41.8 ± 22.1	2.1 (5.3%)	1.000	0.017	0.2 (0.5%)	1.000	0.000
Balance closed Eyes L	Men	54	11.3 ± 13.3	13.0 ± 13.7	1.7 (15.0%)	1.000	0.024	8.4 ± 8.9	11.3 ± 13.6	2.9 (34.0%)	1.000	0.087	−4.6 (−35.2%)	1.000	0.143
	Women	82	8.01 ± 11.9	9.55 ± 15.9	1.5 (19.2%)	1.000	0.033	9.5 ± 12.4	9.0 ± 12.8	−0.5 (−5.2%)	1.000	0.002	−0.1 (−0.9%)	1.000	0.000
Balance closed Eyes R	Men	56	13.2 ± 15.4	12.0 ± 13.1	−1.2 (−9.1%)	1.000	0.013	10.3 ± 13.0	13.1 ± 15.5	2.8 (27.2%)	1.000	0.070	−1.7 (−14.2%)	1.000	0.021
	Women	83	8.32 ± 11.3	9.44 ± 17.3	1.1 (13.5%)	1.000	0.002	8.7 ± 11.9	10.8 ± 18.8	2.1 (24.7%)	1.000	0.020	−0.7 (−8.3%)	1.000	0.004

The number of participants (n) is presented. Within year-1 and year-2, the mean difference for postexercise results is shown in absolute values (and %-of-change) in the columns named: Y1 post-test Y1 pre-2 (%) and Y2 post-test – Y2 pre-test 2 (%), respectively. Here, the *p*-values between the post-test and pre-test 2 within each sex and year are given (analyzed with RM-ANOVA and post hoc test Bonferroni; see methods). In the text, information is given about the few cases in which a significant sex difference occurred in any of the tests performed before and after the exercise intervention in either year. On the right, the mean differences for follow-up results are shown in absolute values (and %-of-change) in the column named Y2 pre 2 –Y1 post (%), with accompanied *p*-values (analyzed with RM-ANOVA and post hoc test Bonferroni; see methods). For each comparison, the partial eta squared effect size (ES) is shown. Y1 pre-test-2 = second pre-test year 1, Y1 post-test = after the 8-week exercise intervention year 1, Y2 pre-test-2 = second pre-test year 2, and Y2 post-test = after the 8-week exercise intervention year 2. 6MWT = 6-min walk test, TUG = timed up and go, HR = heart rate, L = left, R = right.

## Data Availability

The data that support the findings of this study are not publicly available due to privacy reasons but are available from the corresponding author upon reasonable request.
